# Direct Electric Current Treatment under Physiologic Saline Conditions Kills *Staphylococcus epidermidis* Biofilms via Electrolytic Generation of Hypochlorous Acid

**DOI:** 10.1371/journal.pone.0055118

**Published:** 2013-02-04

**Authors:** Elizabeth L. Sandvik, Bruce R. McLeod, Albert E. Parker, Philip S. Stewart

**Affiliations:** 1 Center for Biofilm Engineering, Montana State University, Bozeman, Montana, United States of America; 2 Department of Chemical and Biological Engineering, Montana State University, Bozeman, Montana, United States of America; 3 Department of Electrical and Computer Engineering, Montana State University, Bozeman, Montana, United States of America; 4 Department of Mathematical Sciences, Montana State University, Bozeman, Montana, United States of America; National Institutes of Health, United States of America

## Abstract

The purpose of this study was to investigate the mechanism by which a direct electrical current reduced the viability of *Staphylococcus epidermidis* biofilms in conjunction with ciprofloxacin at physiologic saline conditions meant to approximate those in an infected artificial joint. Biofilms grown in CDC biofilm reactors were exposed to current for 24 hours in 1/10^th^ strength tryptic soy broth containing 9 g/L total NaCl. Dose-dependent log reductions up to 6.7 log_10_ CFU/cm^2^ were observed with the application of direct current at all four levels (0.7 to 1.8 mA/cm^2^) both in the presence and absence of ciprofloxacin. There were no significant differences in log reductions for wells with ciprofloxacin compared to those without at the same current levels. When current exposures were repeated without biofilm or organics in the medium, significant generation of free chlorine was measured. Free chlorine doses equivalent to the 24 hour endpoint concentration for each current level were shown to mimic killing achieved by current application. Current exposure (1.8 mA/cm^2^) in medium lacking chloride and amended with sulfate, nitrate, or phosphate as alternative electrolytes produced diminished kills of 3, 2, and 0 log reduction, respectively. Direct current also killed *Pseudomonas aeruginosa* biofilms when NaCl was present. Together these results indicate that electrolysis reactions generating hypochlorous acid from chloride are likely a main contributor to the efficacy of direct current application. A physiologically relevant NaCl concentration is thus a critical parameter in experimental design if direct current is to be investigated for *in vivo* medical applications.

## Introduction

The treatment of medical implant-associated infections is challenging. A 2004 article in *The New England Journal of Medicine* reported that of 600,000 joint prostheses implanted in the U.S. annually, approximately 12,000 will develop infections [1]. The most common culprits of these infections are staphylococcal species [2]–[7] constituting over 80% of infections associated with medical devices [6], [7]. In a 2011 assessment of the most frequent pathogens in orthopedic infections, *Staphylococcus epidermidis* was identified as the most prevalent bacterial species in both knee and hip arthroprostheses infections, occurring in half of infections documented, followed by *Staphylococcus aureus* (around 20% of cases), and *Pseudomonas aeruginosa* (around 10% and 4% of cases, respectively) [7], supporting reports of coagulase-negative *Staphylococcus* as an increasingly recognized or emerging pathogen in medical device-related infections [7], [8]. These infections are particularly persistent when the bacteria produce a protective matrix and establish as biofilms [9], a bacterial mode of growth known to be significantly more tolerant to antimicrobial agents when compared to planktonic cultures of the same organism. While long-term antimicrobial therapy with multiple antibiotics can be effective in some cases [2], [3], [5], treatment failure may require the removal and replacement of the device in a one or two stage surgical process accompanied with an extensive course of antibiotics [3] and an estimated average cost of medical and surgical treatment of $30,000 [1]. With these consequences, the development of novel strategies for treatment is desirable.

One proposed strategy is the use of direct current to enhance or replace existing antibiotic regimens. The reports of direct current effects vary. Direct current has been demonstrated to have killing efficacy against planktonic bacteria in static and flowing systems [10]–[12] with effects dependent on the electrode material and the medium composition. A 1992 paper used direct current in conjunction with industrial biocides to combat bacterial biofilms and reported current to enhance the activity of several agents [13]. The study reported direct current alone to have little or no effect on the survival of *Pseudomonas aeruginosa* biofilms; however the combination of direct current and biocide was shown to significantly increase killing efficacy of Kathon, glutaraldeyhde, and quaternary ammonium compound. Subsequent investigations studied the use of electric current with antibiotics [14]–[22]. It was proposed that this synergistic phenomenon, termed the bioelectric effect, could be optimized to enhance antibiotic treatment of recalcitrant biofilm infections when antibiotic treatments were minimally effective. The synergy between antimicrobial and current, central to the bioelectric effect, was reproduced in some many studies [14]–[22], with some reporting conditions with a killing effect of current alone [14], [19]–[22], and others concluding current alone to have little or no effect [13]–[15], [17], [18]. Proposed mechanisms for the effect included electrophoretic enhanced transport of antimicrobials through the biofilm matrix [13]–[15], [18], electroporation enhanced uptake of antimicrobials [13], [14], production of gases such as oxygen that increase metabolic and antimicrobial activity [17], [20], and generation of electrolysis products including chlorine [23], [24], hydrogen peroxide [24], and those affecting pH [23], [25]. In other studies, direct current is reported to affect bacterial adherence or stimulate detachment of biofilm to conducting surfaces [23], [26]–[29]. When external electric fields have been applied with insulated electrodes (i.e. no current flow), enhanced bacterial killing by antibiotics [19] or modification of bacterial adhesion [30] has not been observed.

One notable feature of nearly all of the *in vitro* work on the bioelectric effect with antibiotics has been the omission of chloride from the media during electric current exposure [13], [14], [16]–[20], [25]–[29]. This has been done deliberately to preclude the electrolytic generation of chlorine. *In vivo*, however, chloride is abundant and this omission is not relevant. The purpose of the work reported in this article was to investigate the effect of direct electric current against a mature bacterial biofilm under conditions of physiologic saline and a dilute nutrient solution meant to approximate *in vivo* conditions such as those found in an artificial joint. As a model organism we chose *S. epidermidis* but we also present data for gram-negative *P. aeruginosa*. We first sought to determine whether electric current exposure by itself had an effect on bacterial biofilm in the presence of chloride and whether synergy between current and antibiotic could be observed in this milieu. Second, if electric current had an effect on biofilm in the presence of chloride, we sought to establish whether this effect could be attributed to electrolytic generation of hypochlorous acid.

## Methods

### Biofilm Growth

The CDC Biofilm Reactor (Biosurface Technologies Corp., Bozeman, MT, Model CBR90-1DS) is designed to grow repeatable biofilm on polycarbonate disks (called coupons) in a high shear environment. The reactor is a one-liter glass beaker with a side-arm discharge port at approximately 400 mL for effluent drainage by gravity. Eight polystyrene rods, each holding three 1.27 cm diameter polycarbonate coupons, are inserted through the lid, suspending the coupons in the bulk fluid of the reactor. A baffled magnetic stir bar in the center of the reactor provides mixing as well as uniform fluid shear on each coupon. During continuous flow operation, a peristaltic pump is used to pump fresh medium into the reactor through an influent port in the lid. An in-line, glass flow break in the influent feed line prevents back-contamination of the feed carboy. A second port in the lid is attached to a bacterial air vent for gas exchange. A total of 24 coupons are available in the reactor for sampling.

Biofilms were grown on polycarbonate coupons in the CDC Biofilm Reactor using a standard growth protocol for each organism. The *S. epidermidis* growth protocol is analogous to the ASTM E2562-07 “Standard Test Method for Quantification of *Pseudomonas aeruginosa* Biofilm Grown with High Shear and Continuous Flow using CDC Biofilm Reactor” [31] but has been adapted for the main study organism, *S. epidermidis* RP62A (ATCC#35984): The sterilized reactor containing 450 mL of full strength (30 g/L) TSB (tryptic soy broth, Soybean-Casein Digest Medium, Difco) with the effluent tube clamped was inoculated from a frozen stock of *S. epidermidis*. The reactor was placed on a magnetic stir plate in a 37°C incubator and operated in batch with stirring at 125 rpm for 24 hours. After 24 hours, a continuous flow of sterile 1/10^th^ strength TSB was started with a reactor residence time of 30 minutes and overflow drainage to a waste carboy by gravity. Continuous flow ran for 16 hours at the end of which biofilm-coated coupons were ready for use in experimental protocols. It should be noted that the two faces of the coupon (facing inward towards the baffled stir bar versus outwards towards the glass) experience different amounts of shear stress which is a critical factor in biofilm growth. For all experiments the inward face of the coupon was defined as the sample surface. At the end of the growth protocol (just prior to treatment) coupons had a mean LD of 8.46 log_10_(CFU/cm^2^) with a repeatability SD of 0.34 log_10_(CFU/cm^2^) over 25 experiments with 30% of error due to between experiment sources (sampling method follows).

*P. aeruginosa* ERC-1 (ATCC#70088) biofilms were also grown in the CDC reactor with a similar protocol. The medium concentrations, temperature, and timing were the same as those found in the standard method ASTM E2562-07 and differed from the *S. epidermidis* growth protocol as follows: Batch phase was operated for 24 hours with 1/100^th^ strength (0.3 g/L) TSB at room temperature. Continuous flow was run for 24 hours with 1/300^th^ strength (0.1 g/L) TSB at room temperature. At the end of the growth protocol (just prior to treatment) coupons had a mean LD of 8.08 log_10_(CFU/cm^2^) with a repeatability SD of 0.25 log_10_(CFU/cm^2^) over 28 experiments with 96% of error due to between experiment sources (sampling method follows).

### Exposure to Direct Current and/or Antibiotic

**Figure 1:**
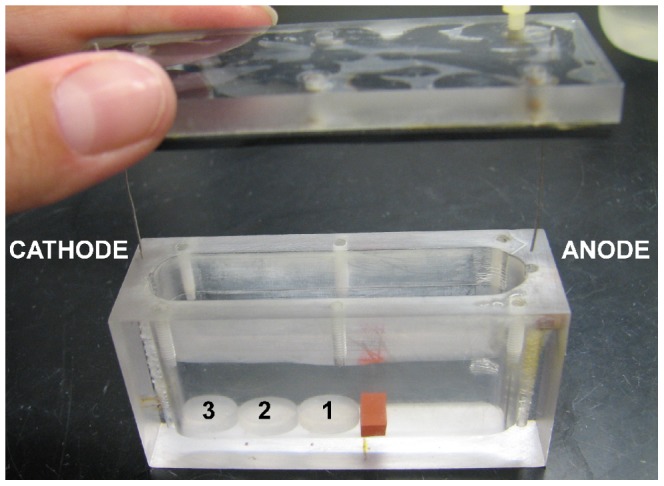
Polycarbonate treatment well. A piece of rubber sheeting on the bottom of the well held the biofilm-coated coupons at the same position (labeled 1–3) for each experiment. Platinum electrodes were inserted through the lid at opposite ends of the well and current was applied lengthwise. The anode was connected to the positive terminal of the power supply and the cathode to the negative.

The treatments were performed in small enclosed wells designed at the Center for Biofilm Engineering, described previously [19]. Inner dimensions were 7.1 cm long by 1.6 cm wide by 3.4 cm tall (Figure 1). Rubber sheeting lined the lid to prevent contamination. Twenty-four gauge platinum electrodes, 3.8 cm long, were pushed through the lid at opposite ends of the wells and extended 2.5 cm from the lid into the treatment well. The anode was connected to the positive terminal of the power supply and the cathode to the negative. A small strip of 3 mm thick rubber sheeting on the bottom of the well, 4.0 cm from the cathode, loosely held the coupons in place so that the positions of the three coupons were the same in all experiments. Each well had a separate circuit with an inline ammeter and current controller (Wavelength Electronics, No. LDD200-1M) on a power supply that generated the direct current. The current controllers maintained a constant current level while voltage fluctuated slightly with changes in the resistive loads across each well.

After growth, biofilm-coated coupons were aseptically removed from the rods of the CDC reactor, dipped in 10 mL of sterile buffered dilution water (42.2 mg KH_2_PO_4_/L, 406.5 mg/L MgCl_2_•6H_2_O/L in reagent-grade water) to remove planktonic cells and placed in sterile treatment wells with the inner face of the coupon (from the CDC reactor) facing up in the well. Twenty mL of a sterile treatment solution (with antibiotic when indicated) was gently added to each well. Direct current was applied across the length of the well.

*Staphylococcus epidermidis* treatments were performed for 24 hours at 37°C. The treatment solution was 1/10th strength TSB with 9 g/L total NaCl (accounting for the salt in TSB) with 2.5 µg/mL ciprofloxacin (Sigma-Aldrich) when indicated. This concentration of ciprofloxacin was approximately 40X the minimum inhibitory concentration (MIC) determined for an aerobic, planktonic culture of the bacteria (data not shown). Direct current levels of 2.0, 3.0, 4.0, and 5.0 mA were applied across the wells with an effective current density of 0.7, 1.1, 1.4, and 1.8 mA/cm^2^, respectively (based on the minimum cross-sectional area of the liquid in the treatment well when coupons were present). Experiments were performed in parallel wells, with and without ciprofloxacin, at each current level. Control wells without current, with and without ciprofloxacin, were run with every experiment. When one current level was investigated, this resulted in four parallel wells corresponding to four treatment levels: (1) a control with no ciprofloxacin and with no current, (2) a well with ciprofloxacin but no current, (3) a well with current but no ciprofloxacin, and (4) a well with both ciprofloxacin and current. In some experiments two current levels were applied for a total of six parallel treatment wells. Three coupons were sampled from exposed wells when only one current level (four wells total) was studied. For experiments with two current levels (six wells total), only the two extreme coupons (positions 1 and 3) were sampled in each well. At least three independent experiments were performed at each current level (*N = *11 experiments total).

While *S. epidermidis* was the primary study organism, treatment of gram-negative bacteria with electric current was also investigated with varying NaCl concentrations. Treatment of *Pseudomonas aeruginosa* biofilms were performed for 20 hours at room temperature. The treatment solution was 1/300^th^ strength TSB (to match continuous flow conditions from the growth reactor) with varying concentrations of NaCl from no added salt (0.017 g/L NaCl from the TSB) to 9 g/L total NaCl. Tobramycin was added when indicated at 10.0 µg/mL tobramycin sulfate (Sigma-Aldrich) to parallel previous bioelectric effect studies using the same concentration [15], [17] or half the concentration (5.0 µg/mL) [14]– of tobramycin against *P. aeruginosa.* The MIC data reported in those studies indicate 10.0 µg/mL tobramycin sulfate to be 10X the MIC for the *P. aeruginosa* strains studied [14]–[17], [19]. Three coupon spacing regimes were used: no spacers, grouped at the cathode (as in the *S. epidermidis* experiments), and an extreme spacing with coupons at the anode, middle, and cathode. For this reason, *P. aeruginosa* results are presented as mean LD for coupons (*n = *2 or 3 coupons per well) from a given treatment well. A total of *N = *14 experiments applied current with and without antibiotic and *N = *5 experiments applied current only, resulting in 34 wells with only current and 16 treatment wells with current and tobramycin. Applied current levels between 0.13 and 2.0 mA (0.05 to 0.71 mA/cm^2^) were restricted by the salt concentration and the range of the current controllers.

### Determination of Biofilm Viable Cell Numbers

At the end of the treatment, coupons were sampled and analyzed as described in ASTM E2562-07 [31] with slight modifications: Treated coupons were gently removed from the treatment well with a sterile hemostat and placed on a sterile surface for sampling. While holding the coupon with the hemostat, the top surface of the coupon was scraped with a sterile wooden stick held perpendicular to the surface for 30 seconds. The stick was swirled in 9 mL of sterile buffered dilution water and then used to scrape the coupon a second and a third time. The coupon was scraped a total of three times for 30 seconds each in this same manner. After swirling the third time, the stick was discarded. The coupon was held over the dilution tube and the scraped surface was rinsed with 1 mL of dilution water to remove any remaining biofilm resulting in a final volume of 10 mL in the dilution tube. The sample was homogenized at 10,000 rpm for one minute to disaggregate biofilm clumps, serially diluted and drop plated using the drop plate method (ten 0.01 mL drops per dilution) on agar. *S. epidermidis* samples were plated on tryptic soy agar (Difco) and *P. aeruginosa* samples were plated on R2A agar (Difco). The colony forming units (CFU) on the plates were counted following overnight incubation at 37°C. A biofilm log density (LD) of viable cells for each coupon was calculated using the mean CFUs counted over 10 drops, the volume of each drop, the surface area of the coupon, the volume of liquid the biofilm was scraped into (the zero dilution), and the dilution at which the colonies were counted: 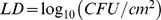 =  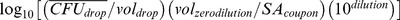. Log reductions (LR) of biofilm cell densities were calculated for each sample based on comparison of the treated sample to the mean of the no-current, no-antimicrobial control (1–3 controls per experiment) within the same experiment: 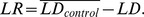



We used a LR per sample, as opposed to a LR per experiment, in order to explicitly model the effect of the location in the well on the mean LR. Either definition of the LR generated the same mean LR, but the SEMs and degrees of freedom for the t-tests based on LRs per sample were larger.

Growth control coupons were sampled directly following the growth protocol each time the reactor was run and sampled in the same fashion. Growth coupons were aseptically removed from the reactor and the coupon holder, dipped in 10 mL of sterile dilution water to remove planktonic cells, and placed on a sterile sampling board. The scraping procedure at this point was the same as above.

### Measurement of Chlorine Generated by Electric Current Exposure

Both biofilm and medium organics can be sources of chlorine demand, which react quickly with available chlorine in the system thereby lowering measurable free chlorine. Consequently, to determine if free chlorine species were generated during the course of electric current exposure, the experiments were repeated without biofilm and without the organics in the medium. The same current levels used in the *S. epidermidis* experiments were applied to 20 mL of a 9 g/L NaCl, 0.25 g/L K_2_HPO_4_ solution equivalent to the salt and buffer concentrations present in the treatment solution. After 24 hours of application, the current was turned off, the lid was gently removed, and liquid samples (0.1 mL each) were pipetted simultaneously at each electrode. Samples were measured by the DPD chlorine method using HACH DPD Free Chlorine Reagent (5 mL Powder Pillows) read on a spectrophotometer at 530 nm. Samples were diluted in reagent-grade water to be within the manufacturer’s range of the reagents. These chlorine concentrations, referred to as “endpoint free chlorine measurements”, were assumed to represent the cumulative amount of reactive chlorine species generated over the 24 hour treatment time.

### Measurement of pH Following Electric Current Exposure

For an estimate of pH changes, the *S. epidermidis* biofilm experiments were repeated without biofilm. The same current levels were applied to 20 mL of the standard treatment solution (3 g/L TSB, 9 g/L total NaCl) at 37°C for 24 hours. For each well, the current was turned off and the pH was immediately measured with a pH meter at each electrode. In a cruder method, pH strips (EMD colorpHast, 0–14 pH range) were spaced at 1 cm spacing and simultaneously dipped into the treatment well while the current was running.

### Chlorine Dosing Experiment to Mimic Electric Current Exposure

In another variation, free chlorine concentrations equivalent to the 24 hour “endpoint free chlorine measurements” for each current level were used to treat *S. epidermidis* biofilms in place of the electric current. For each current level, the previously measured free chlorine concentrations were grouped for both electrodes and averaged. Four free chlorine solutions (corresponding to the four current levels) were made with bleach and sterile laboratory-grade water. Sterile water (no added bleach) was used for the control. Due to the rapid nature of chlorine reactions, the nutrients were added to the chlorine solutions just prior to application to the biofilm as follows. Each free chlorine solution was adjusted to 105% of the target free chlorine concentration. A 20X sterile medium solution was made such that the resulting 1X concentration would be the standard 3 g/L TSB with 9 g/L total NaCl. *S. epidermidis* biofilms were grown in the CDC reactor and transferred to the treatment wells as before. At time zero, 1 mL of the 20X medium solution was added to 19 mL of the given free chlorine solution in a sterile glass vial, swirled, and gently added to the treatment well containing the biofilm-coated coupons. This was done for all five wells. Wells were then treated like the biofilms exposed to current with a 24 hour incubation at 37°C after which biofilms were sampled as usual. No antibiotic was used in this experiment.

### Exposure of Biofilm to Electric Current with Alternate Electrolytes (Omitting Chloride)

In this variation, the electric current was applied to *S. epidermidis* biofilms in treatment solutions without chloride. An electrolyte was still needed for current flow so in place of the 0.154 M Cl^-^ (9 g/L NaCl) in the standard treatment solution, 0.154 M SO_4_^2−^ (as 21.9 g/L Na_2_SO_4_), 0.154 M NO_3_^−^ (as 13.1 g/L NaNO_3_), or 0.154 M PO_4_^−^ (as 10.7 g/L Na_2_HPO_4_ and 9.2 g/L NaH_2_PO_4_ accounting for K_2_HPO_4_ contributions already in the medium) was used with the other components of TSB added individually (at 1/10^th^ strength: 1.7 g/L pancreatic digest of casein (BD Bacto Tryptone), 0.3 g/L enzymatic digest of soybean meal (BD Difco Soytone Peptone), 0.25 g/L dextrose, and 0.25 g/L K_2_HPO_4_). For each alternate electrolyte, applications of 0, 2.0, and 5.0 mA (0, 0.7, and 1.8 mA/cm^2^ respectively) were performed in parallel and all three coupons in the wells were sampled. Other than substitution of the treatment solutions, the growth, treatment, and sampling protocols were the same as the previous *S. epidermidis* experiments with current application. No antibiotic was present in these experiments.

### Staining and Microscopy of Detached Cells and Biofilm

Three wells containing *S. epidermidis* biofilms were treated with 3.0 mA (1.1 mA/cm^2^) of direct current for 24 hours with parallel triplicate control wells without current (as described above). Bulk fluid and biofilm samples were stained using the LIVE/DEAD BacLight Bacterial Viability Kit (Molecular Probes) as well as sampled for viability on TSA. The staining kit contains green-fluorescent SYTO 9 and red-fluorescent propidium iodide to distinguish between cells with intact membranes (“live”) and cells with damaged membranes (“dead”), respectively. One mL samples of the undiluted bulk fluid and a 1∶10 dilution of the bulk fluid (in sterile PBS) were removed from each well and incubated at room temperature with 3 µL of a 1∶1 solution of Component A (SYTO 9) and Component B (propidium iodide) in the dark for 15 minutes. Each stained sample was filtered onto a black polycarbonate membrane (GE Water & Process Technologies, 0.22 µm pore size, 25 mm diameter). The membrane was transferred to a glass microscope slide, a drop of Type FF immersion oil (Cargille) was applied to the filter and a cover slip applied. The samples were visualized with a Nikon Eclipse E800 epifluorescence microscope (100X oil objective, 1.40 NA) with a standard FITC filter (ex: 480/30, DM: 505 LP, em: 535/40) for visualizing green SYTO 9 fluorescence and a standard TRITC filter (ex: 540/25, DM: 565 LP, em: 605/55) for viewing red propidium iodide fluorescence. Paired green and red images were taken at twenty random views (16,389 µm^2^) for each sample using MetaVue software (Universal Imaging Corporation). The area of cell coverage was measured on each digital image using MetaMorph software (Universal Imaging Corporation). A calibration of the measured cell area versus manual cell counts of the digital image was done for 50 clusters ranging in size from 1–32 cells. The calibrated mean area per cell was used to convert the measured area to cell counts for each image resulting in cell counts of “live” and “dead” cells for *n = *20 views for each bulk fluid sample. To complement these measurements, the bulk fluid was also sampled, serially diluted, and plated on TSA for viable cell counts.

Biofilm samples from the same treatment wells were removed, stained on the coupon with LIVE/DEAD solution at a ratio of 3 µL of the 1∶1 SYTO 9 and propidium iodide solution to 1 mL of filter sterilized PBS, and incubated at room temperature in the dark for 30 minutes. Following incubation, the stain was pipetted from the side of the coupon and the sample was gently rinsed with filter sterilized PBS to remove excess stain. The stained samples were then imaged with confocal microscopy or embedded for cryosectioning. Confocal images of the biofilm were taken fully hydrated in a petri plate using a Leica TCS SP5 Confocal (25XW LWD, 0.95 NA). SYTO 9 and propidium iodide fluorescence was excited using 488 nm and 561 nm lasers with detector slits set to 499–551 nm and 579–647 nm, respectively. Images were processed using Imaris software (Bitplane). For cryosectioning, stained coupon samples were cryoembedded in O.C.T. Compound tissue embedding medium (Tissue-Tek) on dry ice as previously described [26]. Five µm thick slices of the embedded samples were cut in a Leica CM1850 cryostat at −20°C and placed on poly-L-lysine coated microscope slides (SuperFrost Plus, FisherBrand). A coverslip was placed over the dry cryosection samples, a drop of water placed on top of the cover slip and the cryosections were imaged using a 60XWI objective (1.20 NA) with the Nikon E800 epifluoresence microscope and filters described above. Green and red images of the cryosections were color combined using MetaMorph software (Universal Imaging Corporation).

### Statistical Methods

Statistical analyses were performed using linear mixed effects (*lme*) models in the nonlinear mixed effects (*nlme*) package [32] in the free statistical and graphing program *R* [33]. The *lme* models accounted for electric current density, position in the well, and salt concentration (*P. aeruginosa* experiments only) as covariates. Antimicrobial treatment, treatment source (electric current versus mimicked electric current for chlorine dosing experiments), and electrolyte were categorical variables. Experiment was a random factor in all models. As unequal variances existed between the no-current controls and current-exposed data (as expected), statistical analyses were performed on LR data sets (compared to the no-current, no-antibiotic control) that were truncated to exclude the controls with antibiotic but no current. The following model selection process was used to determine the most appropriate model for the data. Initially, all two-way interactions amongst the factors were included in the *lme* model. Interactions were investigated by significance tests and interaction plots. Insignificant and unimportant interactions were dropped.

The following models resulted from the selection process. To assess *S. epidermidis* killing effects of direct current in the presence and absence of ciprofloxacin, LRs for each sample were evaluated as a function of antibiotic presence/absence, current density, and position in the well with an interaction between antibiotic and current density. Differential killing across the well was analyzed using a subset of the data as the LR difference between the extreme coupons (coupons 1 and 3) from the same well as a function of current density and antibiotic presence/absence with an interaction term between the two variables. To assess killing of *S. epidermidis* with chlorine doses, LRs for each sample in the chlorine dosing experiment were analyzed as a function of mimicked current density. The chlorine dosing experiment (*N* = 1) was compared to the no antimicrobial, current exposed experiments (*N* = 11) as mean LRs across each well as a function of applied (or mimicked) current density and treatment type (direct current or chlorine dose) with an interaction term between current density and treatment type. Current exposure with alternate electrolytes was analyzed in one model using the nitrate, sulfate, and phosphate data as well as the 2.0 and 5.0 mA data when chloride served as the electrolyte. LR (in comparison to the no-current control of the same electrolyte) was analyzed as a function of current density, electrolyte, and position with an interaction term between current density and electrolyte.

Free chlorine generation was analyzed at each electrode as the free chlorine concentration as a function of current density. Gradients of chlorine across the well were analyzed as the difference in free chlorine concentration between electrodes in the same well (simultaneous samples) as a function of current density.

For *P. aeruginosa* experiments, the following *lme* models were used. To assess the effect of salt on growth, LDs of the no-current controls with and without tobramycin were analyzed separately as a function of salt concentration, and jointly as LDs as a function of antibiotic presence/absence and salt concentration with an interaction term between the two variables. As several spacing regimes were used in the *P. aeruginosa* experiments during electric current treatment, mean LRs across each current-treated well were used as the response. Data from biofilms exposed to current without antibiotic was used to examine current effects at different salt levels. Mean LRs over individual treatment wells were assessed as a function of salt concentration and current density as covariates with an interaction term between the two variables. Tobramycin effects with current at varying salt concentrations were assessed only for experiments with parallel wells with and without antibiotic. Mean LR’s were analyzed as a function of current density, salt concentration, and antibiotic presence/absence. Initially all two way interactions were in the model but nonsignificant interactions between antibiotic and current (p-value = 0.32) and antibiotic and salt (p-value = 0.52) were removed from the model resulting in one interaction term between current and salt for the tobramycin experiments.

The repeatability standard deviation (SD) for lme models was calculated from the R output of within-experiment variance, σ_within_^2^, and between-experiment variance, σ_between_^2^: 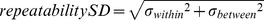. The percent contributions of each source of variance were calculated as the variance for that source divided by the square of the repeatability SD. To maintain a familywise false discovery rate of 5%, the Benjamini-Hochberg procedure was implemented [34]. Thus, hypothesis tests with individual p-values<0.0186 were considered statistically significant.

## Results

### Electric Current Alone Killed *S. epidermidis* Biofilms

**Figure 2:**
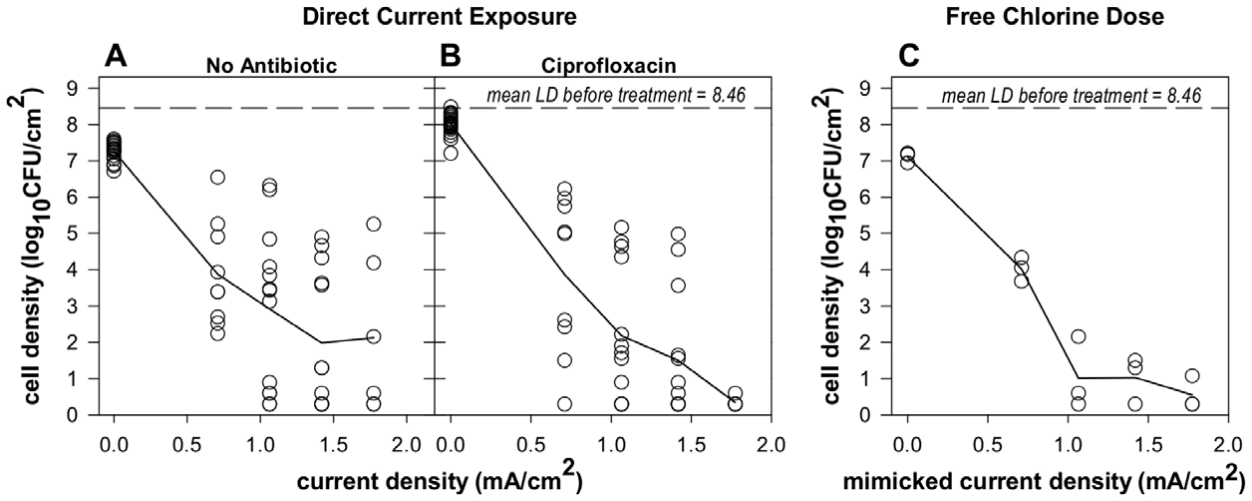
Treatment of *S. epidermidis* biofilm with electric current or a mimicked current exposure using chlorine. Direct current was applied in parallel to wells (A) with no antibiotic and (B) with 2.5 µg/mL ciprofloxacin at one or two current levels for *N* = 11 experiments. (C) An initial dose of free chlorine corresponding to each current level was used to mimic the direct current exposure in the absence of current for *N* = 1 experiment (no antibiotic). All wells contained 3 g/L TSB and 9 g/L total NaCl and treatments lasted 24 hours. Each data point denotes the LD for an individual coupon and lines show connected mean LDs for each current level.

Significant reductions of *S. epidermidis* biofilms were observed when direct current was applied for 24 hours at 37°C at all four current levels (0.7, 1.1, 1.4, and 1.8 mA/cm^2^), both in the presence and absence of ciprofloxacin (Figure 2A and 2B). Data are plotted as individual data points for each coupon sampled. Larger reductions in biofilm viable cell density were observed at higher current levels with mean LRs up to 6.68 log_10_(CFU/cm^2^) at the highest current level (1.8 mA/cm^2^) with ciprofloxacin (p-value<0.0001). The repeatability SD of LR on coupons exposed to current was 1.71 log_10_(CFU/cm^2^) with 24% of variance due to between experiment sources. Statistical analyses were performed based on LR as a function of current level, position, and the presence/absence of antibiotic. A significant linear relationship of increasing LR with increasing current level was observed for wells without antibiotic (p-value = 0.0001) as well as those with ciprofloxacin (p-value = 0.004) demonstrating a dose-responsive effect due to current alone.

### Combining Antibiotic with Electric Current did not Increase Killing of *S. epidermidis* Biofilms

As killing was observed both in the presence and absence of ciprofloxacin, the linear relationships of LR and current level were compared to investigate the role of the antibiotic. No statistically significant difference in killing were observed when 2.5 µg/mL ciprofloxacin was present during direct current exposure (p-value = 0.75). The interaction between current and antibiotic was not significant (p-value = 0.33) indicating that the presence of 2.5 µg/mL ciprofloxacin additionally did not affect the rate of the dose-responsive killing with current level. As the bioelectric effect is discussed as a synergistic increase in killing efficacy when antibiotics are used in conjunction with electric current, an interaction between antibiotic and current would be observed if this type of synergy was present. In these experiments, no statistically significant synergistic or additive responses were observed when 2.5 µg/mL ciprofloxacin was present during direct current exposure.

### *S. epidermidis* Biofilms Closer to the Cathode Experienced Greater Killing that those more Distant

**Figure 3:**
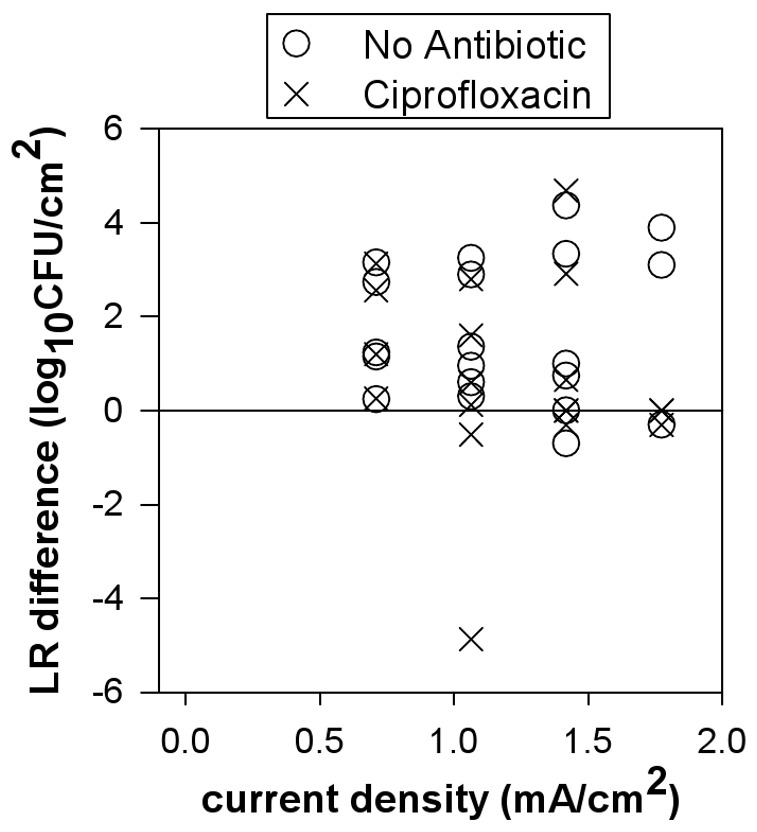
Increased killing of *S. epidermidis* biofilm adjacent to the cathode. Within each well, a trend of larger reductions at coupon 3 (adjacent to the cathode) compared to coupon 1 (near the center of the well) was observed following electric current treatment, as indicated by positive values. Each symbol denotes the measurement of the difference in LR between the extreme coupon positions (coupon 3 minus coupon 1) in a current-exposed well.

An unexpected but significant observation was the relationship between log reductions and the position of the sample in the treatment well during current exposure (p-value = 0.0005). To determine if a definitive killing trend existed across the treatment wells, the difference in LR for the coupon at the cathode (coupon 3) with respect to the coupon near the center of the well (coupon 1) from the same current exposed well was examined for the *S. epidermidis* experiments (Figure 3). Non-zero differences would indicate differential killing due to position. Five of the thirty-nine wells exposed to current had total kill (below detection limit) on both coupons and thus had a position difference of zero. In 28 of the 34 remaining experiments, larger reductions in bacterial density were observed at coupon 3 compared to coupon 1 as indicated by a positive LR difference. These LR differences between the extreme coupons did not change significantly with current level (p-value = 0.71) or the presence or absence of ciprofloxacin (p-value = 0.13) but the pooled difference across all wells was significantly positive on the average (p-value = 0.0003). These data thus indicate a trend of increased killing at the cathode (coupon 3) compared to samples in the center of the well when direct current is applied. When no current was applied (control samples), position did not have a statistically significant effect.

### Electric Current Resulted in the Generation of Free Chlorine

**Figure 4:**
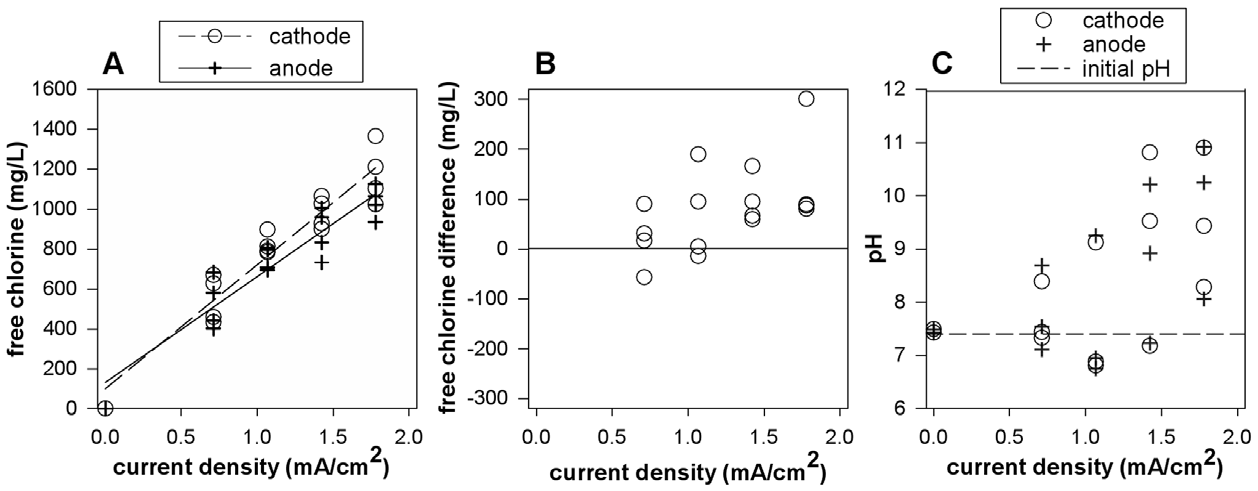
Chlorine generation and pH changes with electric current in the absence of biofilm. (A) Free chlorine was measured at each electrode after 24 hours of current when the direct current exposures were repeated without sources of chlorine demand (biofilm and organics in the medium). These “endpoint free chlorine concentrations” approximate the cumulative chlorine generated over the course of treatment without reaction. (B) The free chlorine differences between electrodes (cathode minus anode) in each well indicated a trend of higher free chlorine concentrations at the cathode as indicated by positive values. (C) pH measurements were taken at each electrode after direct current was applied for 24 hours to the standard treatment solution (with organics). Symbols indicate individual measurements and lines (A) show linear mixed effects models.

Due to the chloride in the medium, chlorine generated from electrolysis was suspected as a possible etiologic agent for observed reductions in biofilm density. During electric current exposure, free chlorine would be generated continuously but the available chlorine would react quickly with biofilm and the organics in the medium and was not measurable when those sources of chlorine demand were present. Therefore, the direct current exposures were repeated without the sources of chlorine demand (biofilm and organics) and sampled for chlorine at 24 hours. These measurements are referred to as “endpoint free chlorine concentrations” and approximate the cumulative chlorine generated over the course of treatment without reaction. Significant concentrations of free chlorine were generated at all four current levels over 24 hours (Figure 4A). Concentrations ranged from 400–680 ppm free chlorine at the lowest current level (0.7 mA/cm^2^) to 930–1400 ppm free chlorine at the highest current level (1.8 mA/cm^2^). A strong linear relationship was observed with increasing free chlorine with increasing current levels (p-value<0.0001 for anode samples as well as cathode samples). A significant gradient of free chlorine was also observed across the well (Figure 4B). On average, free chlorine concentrations were higher at the cathode compared to the anode within the same well (p-value = 0.012). Any changes in the magnitude of the chlorine difference with increasing current levels were not significant at the comparison value (p-value = 0.038). When these same experiments were performed with the organics in the solution, free chlorine was undetectable at 24 hours. It is important to note that these chlorine concentrations are cumulative and would be nowhere near as high at any given time during electric current treatment when biofilm and organic sources of chlorine demand are present.

### Electric Current Resulted in pH Changes

pH measurements were taken after 24 hours of direct current application to the treatment solution (3 g/L TSB, 9 g/L total NaCl, no biofilm) to investigate an overall change in pH as well as pH gradients across the well. After 24 hours, the current was turned off and measurements were immediately taken at each electrode with a pH meter (Figure 4C). Slight increases in pH were generally observed after current was applied, however the increases were not statistically significant when compared to the initial pH (p-values = 0.059 and 0.062 for measurements at the anode and cathode, respectively). Comparisons of these measurements within each well were inconsistent with respect to pH being higher at one electrode over the other and no statistically significant difference was observed that would indicate a pH gradient across the wells (p-value = 0.97). However, to validate these observations, pH measurements were taken with pH strips while the current was running. At five minutes, pH gradients around 3 at the anode and 9 at the cathode were observed at the four current levels studied with values between 6 and 7 at the center of the wells (data not shown). Any pH gradient across the well would be expected to rapidly dissipate when current was stopped due to diffusive and convective mixing. These observations indicate a large pH gradient across the wells when the current is running, but no net change in the pH of the solution.

### Free Chlorine Addition in the Absence of Electric Current Mimicked Biofilm Killing by Current

To investigate the contribution of chlorine generation to direct current effects, the following experiment was performed to determine if a free chlorine dose, representative of the concentrations measured when sources of chlorine demand were absent, could be used to mimic direct current exposure. For each current level, the mean endpoint free chlorine concentrations for both electrodes (Figure 4A) were used to determine the treatment concentration of free chlorine to mimic the direct current effect. The averaged concentrations were 0, 560, 730, 920, and 1,120 ppm free chlorine for 0, 0.7, 1.1, 1.4, and 1.8 mA/cm^2^, respectively. Because the nutrients present a very large source of chlorine demand, chlorine solutions were made in water and the nutrients were added immediately preceding application of the chlorine-nutrient solution to the biofilm samples for a 24 hour exposure. This approach accounted for simultaneous chlorine reactions with the biofilm and the organic medium components. Significant reductions in cell density were observed with chlorine doses at all levels (Figure 2C). The reductions followed a dose-responsive pattern with a linear trend of increasing LR with increasing mimicked current level (i.e. chlorine dose, p-value = 0.003). When the mimicked current with chlorine dosing (*N* = 1 experiment) was compared to direct current exposure (*N* = 11 experiments, Figure 2A) as a function of current density (or mimicked current density), no statistically significant difference was observed between treatment with current versus mimicked current with the chlorine dosing (p-value = 0.98), nor was there a significant difference in the LR trend over current (p-value = 0.76). While dose-responsive biofilm reductions with increasing chlorine doses would be expected, the specificity of the applied dose to each current level to generate similar reductions demonstrates more than a partial contribution to efficacy. These results indicate that chlorine generation plays a predominate role in direct current effects in this system but additionally do so in a dose-responsive manner that is based on the current level applied and subsequent amount of free chlorine produced.

### Electrolytes other than Chloride Produced Less Biofilm Killing by Electric Current

**Figure 5:**
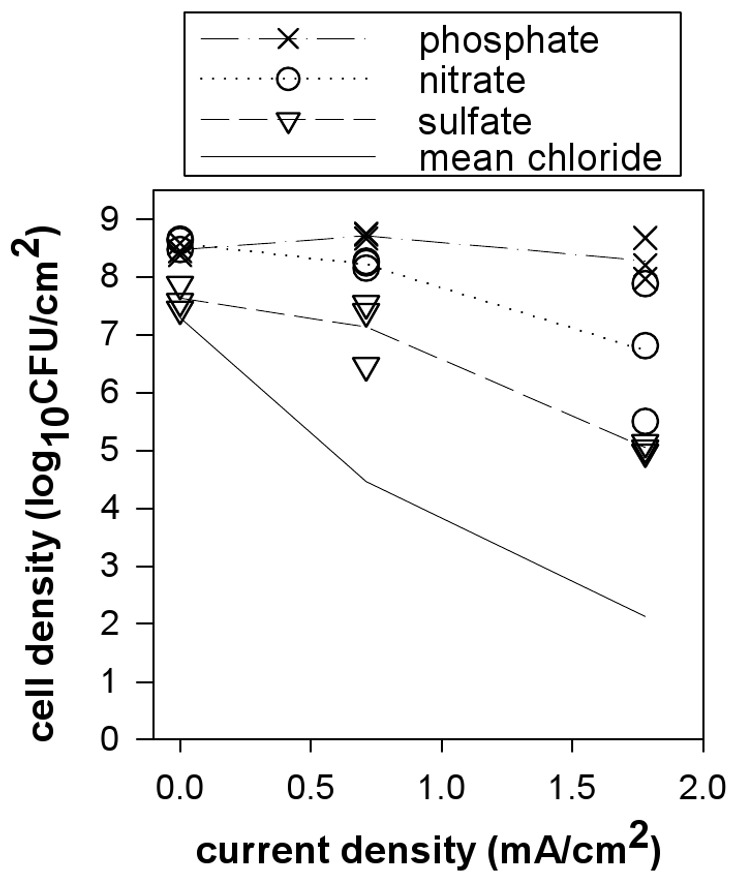
Treatment of *S. epidermidis* biofilm with electric current using various electrolytes. Direct current was applied to biofilms for 24 hours. The treatment solutions had all of the components of TSB except NaCl. Chloride was replaced with nitrate, phosphate, or sulfate at the same molarity (0.154 M) used for chloride experiments. The mean chloride results for 2 and 5 mA are shown for comparison (full data set in Figure 2A). Each symbol indicates the LD for an individual coupon and lines show the connected means for each current level.

Indications of the importance of chlorine generation in direct current efficacy led us to question if electric current effects would be observed without chloride in the system. In the next variation, current was applied to *S. epidermidis* biofilms in the absence of chloride using alternate electrolytes in the treatment solution (Figure 5). Sodium chloride is not only an electrolyte but also a parameter affecting growth of *S. epidermidis* and thus an ideal electrolyte was difficult to determine. As other halide salts would generate similar biocidal products, nitrate, sulfate, and phosphate were used as electrolytes in these experiments. Growth curves of planktonic cultures in the three alternative electrolytes solutions versus the chloride containing solution were similar during exponential phase but final log densities were half to one log order lower than those with chloride (data not shown). Thus, for these experiments, biofilms were grown in the CDC reactor using the standard growth protocol (with chloride) and the application of the alternative electrolyte solutions (and subsequent removal of chloride) only occurred during current application. Controls without current were run in parallel to account for any effects of the alternative electrolyte on biofilm growth or survival over the treatment time. The lowest and highest current levels from the chloride experiments were applied to each solution for 24 hours (Figure 5). Reductions in biofilm were observed with direct current application when sulfate or nitrate served as the electrolyte with a statistically significant dose response of LR with current level for sulfate (p-value = 0.013) but not for nitrate (p-value = 0.066). Reductions were larger for electric current exposure with sulfate as the electrolyte (a 3-log mean reduction at the highest current density) compared to nitrate (a 2-log mean reduction at the highest current density), however, neither showed as strong of an effect as the 5-log reduction seen at the highest current level when direct current was applied with chloride present. In contrast, when phosphate was used as the electrolyte, the application of electric current at the same current levels had no effect on biofilm viability (p-value = 0.58).

### Observation of a Paradoxical Increase in *S. epidermidis* Biofilm Cell Numbers with Antibiotic Treatment

Control treatment wells (one without antibiotic, one with ciprofloxacin) were run in parallel with every experiment and received no current for the duration of the 24 hour treatment. The mean LD for no-current controls without antibiotic was 7.24 log_10_(CFU/cm^2^) with a repeatability SD of 0.27 log_10_(CFU/cm^2^) with variance mainly due to between experiment sources (85%). The mean LD for no-current controls with ciprofloxacin was 8.00 log_10_(CFU/cm^2^) with a repeatability SD of 0.28 log_10_(CFU/cm^2^) with variance mainly due to between experiment sources (83%). The LR due to ciprofloxacin in the no-current controls was statistically significant (p<0.001). We are 95% confident that the *increase* in log density when ciprofloxacin is present is between 0.66 and 0.89 log_10_(CFU/cm^2^). While this seems contradictory to the action of an antibiotic, in this system it can be explained by differential detachment of cells from the coupon surface over the course of the 24 hour treatment. The mean LD on coupons at the start of the 24 hour treatment was 8.46 log_10_(CFU/cm^2^) so LDs decrease over the course of treatment in both control types. We hypothesize that in the presence of ciprofloxacin, more cells remained attached to the coupon surface versus detaching into the harsher environment of the bulk fluid containing the antibiotic. In contrast, detachment may have been less inhibited in the absence of antibiotic. This observation is supported by the fact that control wells with no current and no antibiotic were typically turbid after treatment while the corresponding no-current wells with ciprofloxacin were not. The absence of any killing efficacy of the antibiotic against biofilm bacteria shows that this model system captures the remarkable tolerance of biofilm towards antibiotics.

### Cell Detachment was Observed in Control and Electric Current Treated Samples

Biofilm detachment was assessed with viable cell counts on TSA and LIVE/DEAD staining of the bulk fluid and biofilm following exposure to 3.0 mA (1.1 mA/cm^2^) of direct current for 24 hours. The LIVE/DEAD BacLight kit uses two stains to distinguish between cells with intact membranes (green “live” cells) and cells with damaged membranes (red “dead” cells). While membrane integrity is an important parameter in cell viability, green staining of cells is not a strict parameter for live cells nor does red staining strictly indicate non-viability, thus plate counts and staining are complementary methods. Viable cells in the bulk fluid where considerably higher in the no-current control wells with a mean LD +/− SD of 8.01+/−0.09 log_10_(CFU/mL) compared to 1.15+/−0.78 log_10_(CFU/mL) viable cells in the bulk fluid of the current exposed wells. Total cell counts of filtered bulk fluid samples were calculated from the combined number of stained cells (“live” and “dead”) via microscopy. Detached total cells in the bulk fluid after 24 hours of treatment were 1-log higher in control wells with mean log total cell counts +/− SD of 8.72+/−0.01 log_10_(cells/mL) in control wells and 7.67+/−0.45 log_10_(cells/mL) in the current exposed wells; however the ratios of green “live” or red “dead” cells to total cells in the images showed 92% “live” cells in the bulk fluid of control wells (8.68+/−0.03 log_10_(“live” cells/mL)) compared to only 2% “live” cells of the total detached cells in the wells exposed to 3.0 mA direct current (5.40+/−1.12 log_10_(“live” cells/mL)).

**Figure 6:**
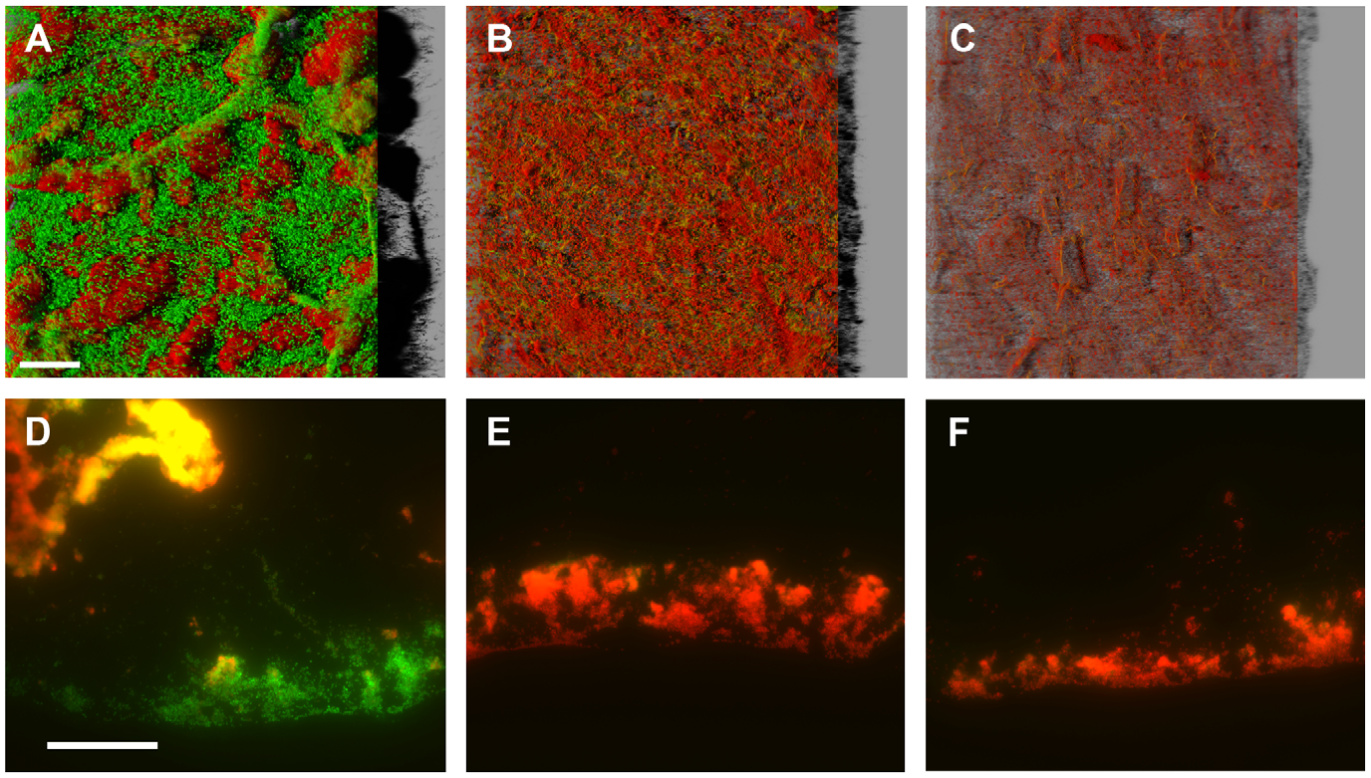
Images of *S. epidermidis* biofilm after 24 hours of no current and 3 mA direct current exposure. Biofilms are stained with LIVE/DEAD BacLight stains. Green color indicates cells with intact membranes (“live” cells) while red color indicates cells with damaged membranes (“dead” cells). (A ) Confocal and (D) cryosection images of the control biofilm. Confocal and cryosection images of biofilm exposed to 3 mA of current are shown for position 1 (B and E) and position 3 (C and F) from the same treatment well. The scale bar for confocal images is 100 µm. The scale bar for cryosection images is 50 µm.

LIVE/DEAD stained biofilm samples from the same treatment wells were imaged with confocal microscopy and cryosectioning. The topography of the unexposed control biofilms was very heterogeneous with many columns, towers, and streamers characteristic of a mature biofilm (Figure 6A and 6D). The majority of distinguishable cells stained green although intermittent distinct red cells were found with searching in both the confocal and cryosectioned images. Broader red staining was also observed on the surface of tower structures. In contrast, electric current treated biofilms were thinner and flatter with the majority of distinguishable cells staining red, indicating damaged cells, for biofilm samples at the center of the well at position 1 (Figure 6B and 6E) as well as adjacent to the cathode at position 3 (Figure 6C and 6F). Some broad red staining and sparse green staining was observed in areas of clustered red cells and seemed to be associated with loose matrix material as distinct green cells were not observed. Biofilm samples from position 1 in the center of the well were visually observed to be slightly denser than those at the cathode with more observations of dim green with red cell clusters. Biofilm samples from position 3 near the cathode showed mainly red cells that ranged from dense clusters of red cells to areas of sparse biofilm only several cells thick. This reduced topography is pronounced considering all biofilm samples (control and treated) came from the same growth reactor and at time zero were mature biofilms resembling the control biofilm.

These biofilm and bulk fluid measurements can be used to assess the components of observed biofilm reductions due to detachment versus killing. Detached cells were found in the bulk fluid of the treatment wells, however more viable cells, total cells (“live” and “dead”), and “live” cells were observed in control wells compared to the wells with electric current indicating that, while biofilm detachment occurs, it is not a treatment specific event. “Dead” stained biofilm was observed still attached to the surface of coupons following electric current exposure although it was reduced in thickness and visually seemed to have decreased total cell density per volume biofilm. During the dose-response experiments described previously, electric current treated biofilms were observed to be looser than controls when sampling for viability. These observations may indicate detachment mechanism and thus an assessment of the LR components (killing versus detachment) should be considered. Due to the heterogeneity of thickness and density of the biofilm samples, quantitative estimates of LR based on biofilm structure in the microscope images would be very difficult but the following hypothetical scenarios may aid in interpretation. If a homogeneous slab biofilm is assumed and the effect of electric current exposure is proposed to only cause biofilm detachment (i.e. no effects on viability), a 2/3 reduction in biofilm thickness would only represent a 0.5 LR of biofilm. If, in addition, the treatment was hypothesized to affect matrix structure such that the number of cells per volume in the remaining slab biofilm was reduced, for example, to 25% of the cells per biofilm volume in the untreated control, the combined decrease in thickness and density would only represent a 1.1 LR. While these values are hypothetical, the detachment and thinning scenarios cannot completely explain the 4.34 log_10_(CFU/cm^2^) mean LR observed at 3.0 mA (1.1 mA/cm^2^) with no antibiotic (Figure 2A), indicating a large effect due to killing. Together, these results suggest that electric current exposure prevented the growth of and killed biofilm and planktonic phase bacteria. While some detachment occurred during electric current treatment and was observed to impact biofilm structure, the predominant effect on the biofilm was loss of viability.

### Electric Current has a Killing Effect on *Pseudomonas aeruginosa* Biofilms

**Figure 7:**
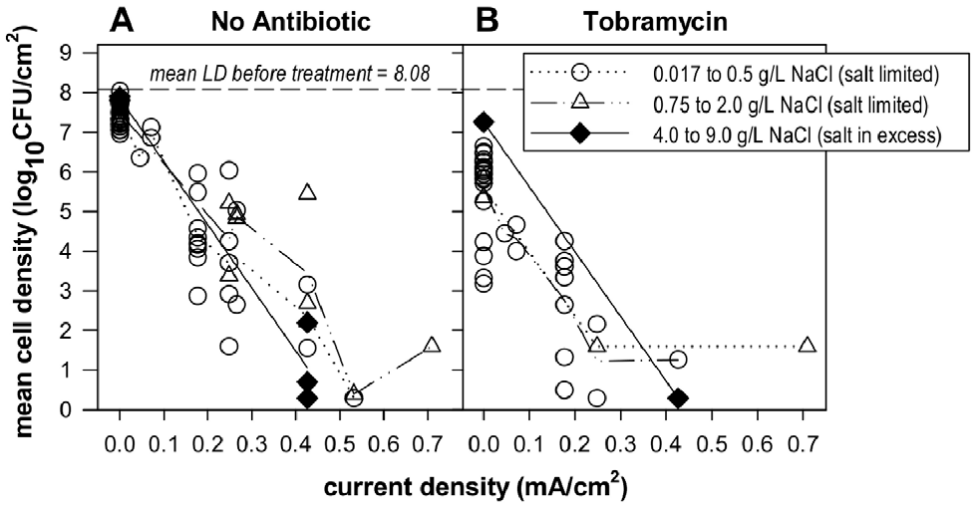
Treatment of *P. aeruginosa* biofilm with direct current. Direct current was applied to biofilm in wells containing (A) no antibiotic and (B) 10 µg/mL tobramycin sulfate for 20 hours. All wells contained 0.1 g/L TSB with varying amounts of total NaCl. References to theoretical salt limitation or excess salt apply only to wells with current. Each symbol indicates a mean LD on coupons across one well and lines show connected means for each current level.

A dose-responsive killing effect of current was observed against gram-negative *P. aeruginosa* biofilms over a twenty hour treatment (Figure 7). Each data point in Figure 7 is the mean LD across coupons (*n = *2 or 3) sampled from the same treatment well in the same experiment. The standard errors (SE) associated with each data point (i.e. the SE of the mean LD of the coupons in each well exposed to current over 50 wells in *N = *19 experiments) were between 0.05 and 1.75 log_10_(CFU/cm^2^) with the exception of eight wells in which all samples were below the detection limit and thus had no variability. Most of this within-experiment variability was observed for direct current levels of 0.18–0.43 mA/cm^2^ for no-antibiotic wells and 0.07–0.43 mA/cm^2^ with tobramycin while standard errors less than 0.32 log_10_(CFU/cm^2^) were observed outside these ranges. In these experiments, TSB was again used as the nutrient in the treatment solution but at 3% of the concentration that was used in the wells during treatment of *S. epidermidis* biofilms and thus represents a system with lower chlorine demand from organic medium components.

Experiments were first analyzed for the effect of the salt concentration during electric current exposure. While the sodium chloride levels are grouped in ranges of concentrations in Figure 7 for clarity of presentation, salt concentration was used as a covariate for analysis. In the absence of current, the salt concentration in the control wells over the 20 hour treatment had no significant effect on the LD in control wells (p-value = 0.12) and was determined not to affect biofilm growth or survival during the treatment time. The mean LD of the no-current, no-antibiotic controls across salt levels was 7.50 log_10_(CFU/cm^2^) with a repeatability SD of 0.34 log_10_(CFU/cm^2^) with 66% of variance due to between experiment sources. For any well with current, two boundaries for chlorine were calculated: the maximum free chlorine concentration if all the chloride in the treatment solution was converted to free chlorine, and the theoretical chlorine concentration if all the current that runs through the treatment solution goes to reactions that generate free chlorine. Experimental parameters where the theoretical maximum chlorine concentration based on current level exceeded the available chloride in the system were defined as theoretically “salt limited”. The mean LRs were analyzed as a function of current density and salt concentration for the no-antibiotic data. Figure 7A indicates that the mean LD of organisms on the coupons decreased as direct current increased with the same rate regardless of salt concentration. The increasing LR with current level was statistically significant (p-value = 0.008) but was not found to be affected by the salt concentration (p-value = 0.72) and no significant interaction was observed between the two factors (p-value = 0.83). The repeatability SD of mean LR for wells exposed to current was 1.35 log_10_(CFU/cm^2^) with 100% of variance due to within experiment sources. As the rates of biofilm reductions with current level were the same across the salt concentrations, it was concluded that the treatment wells had sufficient chloride available and were thus not “salt limited” in the range studied. Looking back at the chlorine generation measured for the *S. epidermidis* current levels (all of which had excess salt available), the mean endpoint free chlorine measurements across each well (Figure 4A) were only 12–21% of the theoretical maximum free chlorine level that would be achieved if all current flow went to the generation of free chlorine. If the type of electrolytic “efficiency” seen with excess salt also applied to the salt and current parameters in the *P. aeruginosa* experiments, the actual chloride required in the medium may be much lower than the theoretical extremes used to define salt limitation, providing a possible explanation for the lack of NaCl effects at the experimental conditions studied.

The effect of tobramycin with varying salt concentrations and current densities was then analyzed (Figure 7B). In the absence of current, biofilms treated with tobramycin had a significant mean LR of 1.92 log_10_(CFU/cm^2^) compared to no-current, no-antibiotic controls (p-value<0.0005) with a repeatability SD of 0.78 log_10_(CFU/cm^2^) with no change in LR due to the salt concentration (p-value = 0.45). When current was applied, a significant linear trend of increasing biofilm reductions with increasing current was again observed (p-value = 0.001). A statistically significant increase in LR of 1.70 log_10_(CFU/cm^2^) was observed when tobramycin was present in wells with current compared to wells without antibiotic (p-value = 0.001), however the interaction between tobramycin and current level (which would indicate synergy between the antibiotic and electric current) was not significant (p-value = 0.33). The repeatability SD of mean LR for wells exposed to current was 1.17 log_10_(CFU/cm^2^) with 100% of variance due to within experiment sources. The tobramycin effect during current application is very close to that seen in the no-current controls and the lack of interaction with current level fails to demonstrate synergy between current and tobramycin. Thus, in this system the killing by tobramycin seems to be a separate, additive effect that is not enhanced by the application of current.

## Discussion

The purpose of this study was to investigate the application of direct current under conditions physiologically relevant to the milieu of an infected artificial joint. While past biofilm experiments in this area of research have typically used a chloride-free solution to minimize electrolysis effects, in this system a salt concentration of normal saline (0.9% NaCl) was used in order to mimic the physiologically relevant environment.

Our results are consistent with electrolytic generation of hypochlorous acid, a potent disinfectant, at the anode leading to biofilm killing. The half-reactions for electrolysis of a sodium chloride solution are:
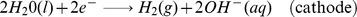


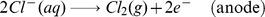



Chlorine gas has limited disinfection power however when generated in water, produces reactive free chlorine compounds including hypochlorous acid (HOCl) and hypochlorite (OCl^-^):
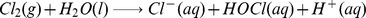


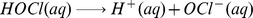



When combined, the overall electrolysis reaction is:
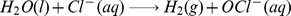



Although the overall reaction would result in no net pH change, the cathodic and anodic reactions indicate localized pH decreases at the anode and increases at the cathode consistent with our observations.

This mechanism of electrolytic generation of chlorine in the presence of aqueous chloride is consistent with the following experimental observations: 1) Killing effects associated with direct current exposure were observed against *S. epidermidis* and *P. aeruginosa* biofilms in the absence of antibiotic (Figure 2A and 7A). 2) Biofilm reductions were dose responsive with increased killing observed with increasing current density (Figure 2A and 2B and Figure 7). Increasing the current density is expected to increase the rate of chlorine generation. 3) High concentrations of free chlorine were measured in the system when exposed to electric current (Figure 4A). The amount of chlorine generated also follows a linear dose response with current density. 4) The effect of electric current was accurately mimicked by dosing of an equivalent concentration of chlorine (Figure 2C). 5) Less killing was observed during electric current treatment at the same current levels when nitrate and sulfate were used in place of chloride during current exposure (Figure 5). 6) No killing effect was observed when current was applied at the same levels when phosphate was used in place of chloride. 7) Biofilm killing was greater on the coupon immediately adjacent to the cathode where higher chlorine concentrations were also observed (Figure 3). We conclude that electrolytic generation of chlorine is likely to be important *in vivo* where chloride ion is naturally and invariably present.

The bioelectric effect is typically discussed as a synergistic effect between biocide and direct current when either alone would have minimal to no impact. There was no effect of ciprofloxacin in the *S. epidermidis* experiments (Figure 2A and 2B). The reduction due to tobramycin in *P. aeruginosa* experiments (Figure 7) was comparable when current was present or absent and determined to be an additive effect, independent of the presence of direct current. Thus, the results reported here do not conform to the bioelectric effect model as we find substantial killing by current alone and no synergistic enhancement of killing provided by combining current and antibiotic.

Electrolytic generation of chlorine explains the direct current efficacy observed in this study when the experiment accounted for physiologic saline conditions (9 g/L NaCl). Doses of chlorine alone at concentrations of 25 to 100 mg/L have been reported to partially kill or remove *S. epidermidis* [35], [36] or *S. aureus* biofilms [37] and those results are furthermore confirmed in this study. Considered a rapid speed kill agent, chlorine works as a disinfectant by oxidizing organic material [38]. Active against many organisms, chlorine and hypochlorite salts are the most common disinfectant in municipal water treatment facilities [39]. HOCl is also produced in the phagolysosomes of human neutrophils from Cl^-^ and H_2_O_2_ by myeloperoxidase to kill and digest foreign microbes for immune defense [40]–[42]. Although seemingly supportive of chlorine in the body, HOCl is isolated to the phagolysosomes within these cells and release of chlorine species from ruptured neutrophils has been implicated in host tissue damage [40] although host systems to prevent HOCl tissue damage are also reported [43]. HOCl reacts with a number of cellular targets [44] demonstrating broad reactivity with bacterial and host cells [45]. Electrolytic generation of chlorine gas in an aqueous solution is a means of generating this potent disinfectant. Although chlorine would be generated at the anode, free chlorine was found throughout the system due to convective mixing (Figure 3A). However, due to the highly reactive nature of chlorine, the free chlorine would remain very low during the course of treatment and may be difficult to detect when chlorine demand is high in the system. Chlorine was not detected over the course of 7 days of electric current in a recent study with 0.5 g/L NaCl from TSB [46]. In our system, chlorine was only detected when the organics were removed from the system.

The speciation of free chlorine is pH dependent as predominantly hypochlorous acid (HOCl) below pH 7.5 and hypochlorite ion (OCl^-^) above. Hypochlorous acid is known to be 100 times more reactive than the hypochlorite ion and a stronger disinfectant. In the study reported here, a pH gradient around 3 at the anode to 9 at the cathode was observed across the treatment wells when the current was running. While the pH gradient alone could have substantial effects on bacterial viability, it may also affect chlorine speciation and efficacy. Insight into the effects of pH versus chlorine can be drawn from emerging use of electrolyzed water (EW) as a disinfectant in the food industry. EW is typically generated from the electrolysis of dilute sodium chloride solutions that is then collected at each electrode for subsequent application with hypothesized action due to pH, chlorine, and the oxidation-reduction potential (ORP) of the solutions. A recent comparison of acidic EW and basic EW treatment of *S. aureus* biofilms by Sun et al. demonstrated high killing efficacy but no removal of biofilm with acidic EW solutions but high removal and no killing of biofilm with basic EW [47]. The removal effect was partially reproduced by increasing the pH without EW but representative decreases in pH did not reproduce the killing effect indicating other EW properties, such as chlorine or oxidative properties, to be responsible for killing efficacy. In other studies, reductions by previously electrolyzed acidic EW against planktonic *E. coli* and *L. monocytogenes* were reproduced with a free chlorine concentration close to those found in the respective EW solution [48]. In our study, we used non-electrolyzed free chlorine solutions to effectively mimic direct current. Reductions were very similar when chlorine doses were used indicating chlorine as a major factor although pH may affect localized changes across the treatment well and ORP might be an additional contributing factor.

The significance of chloride in the system was further demonstrated when the same current levels were applied without chloride in the system. No electric current effect was seen when *S. epidermidis* biofilms were treated with phosphate serving as the electrolyte. Intermediate 2-log and 3-log mean reductions were observed at the highest current level when nitrate or sulfate served as the electrolyte but neither had as strong of an effect as the 5-log mean reduction when chloride was present in the system (Figure 5). The effects with sulfate and nitrate are hypothesized to be due to electrolysis reactions and products other than those generating chlorine. Davis et al. found similar results with electrolyte variations in the medium during direct current treatment of planktonic *E. coli* cultures [12]. They reported an initial 8-log planktonic cell culture to be very minimally reduced when phosphate was used in the medium, a 1-log reduction with sulfate, a 2-log reduction with nitrate, but no survival when chloride was present over a 4 hour treatment with 400 µA across a 10 mL culture. In studies of electrochemical disinfection of wastewater in a plug flow reactor, Li et al. observed reduction efficiencies of *E. coli* when wastewater contained NaCl but no disinfection efficacy was observed when water contained NaNO_3_ or Na_2_SO_4_ [49]. Wattanakaroon and Stewart reported a 4-log increase in *Streptococcus gordonii* biofilm killing when 2 g/L NaCl was added to the solution during electric current treatment although also reported extensive corrosion of the stainless steel electrodes with chloride present [21]. Sun et al. reported that while acidic electrolyzed water generated from the electrolysis of a 0.1% NaCl solution had strong killing efficacy against *S. aureus* biofilm, the same pH electrolyzed water generated from a 0.1% NaNO_3_ solution had minimal efficacy [47]. These observations show chloride as an important parameter demonstrated to increase killing efficacy of electric current.

Another point of consideration is electrode material. Davis et al. reported the electrode material to be a critical parameter in studies of electric current application to planktonic cultures [11]. With a gold cathode, anodes made of carbon or platinum were reported to be the most effective while silver, nickel, or copper corroded to the point of breakage during experiments. In other studies, the release of metals from the electrodes has been intentionally used to deliver silver for treatment [50], [51]. Stainless steel has predominantly been used for both electrodes [13], [14], [17], [18], [20]–[23], [46], [52], as one electrode alternating polarity throughout exposure as anode or cathode [13], [15], or as the cathode [27], [29], [53] in studies investigating electrical effects on biofilm although use of platinum [16], [25], and graphite [22], [46] are also reported. In some of these studies, corrosion or discoloration has been reported as an observation with stainless steel electrodes. We also began our study with ferrous electrodes (no data shown). These electrodes experienced significant corrosion and precipitation of colored corrosion products. These products were observed to have lethal effects in our system which lead to the use of platinum electrodes. Recent studies have also linked the use of stainless steel electrodes to both corrosion products and higher bacterial reductions when compared to graphite electrodes [22], [46]. It has been suggested that use of stainless steel electrodes may have contributed to the larger reductions reported in previous studies of the bioelectric effect [22]. These types of observations might be difficult to detect in a flowing system.

So how do our findings relate back to treatment of an infection *in vivo*? In the past, the bioelectric effect has been suggested as a way to enhance antibiotic treatment of infections. Our *in vitro* results suggest a mechanistic basis for the efficacy of electric current alone when chloride is present. This may be part of the explanation for antimicrobial efficacy of electrical treatments reported in prior studies in animals. Successful electrical current treatment of *S. epidermidis* has been demonstrated in a goat model along surgical pin tracts connecting an external fixation frame to bone for use in reconstructive bone surgery with the pin acting as an electrode [54]. The anode, which would be the site of generation of hypochlorous acid, was a platinum ring contacting the skin with the stainless steel fixation pin serving as the cathode. Secinti et al. used titanium and silver coated titanium screws as anodes to study the intentional release of silver particles to treat *S. aureus* on vertebral implants in rabbits [55]. While both electrically treated screws decreased cell counts, release of titanium was associated with significant bone tissue inflammation where none was reported with silver. Del Pozo et al. showed killing efficacy of electric current application against *S. epidermidis* on stainless steel implants in the tibia of rabbits without antibiotic over a 21 day exposure although discoloration of the bone was also noted [56].

Here we have reported killing efficacy of direct current against *S. epidermidis* and *P. aeruginosa* biofilms and conclude these effects to be due to electrolytic generation of free chlorine. Localized generation of an antimicrobial at the site of infection is an attractive strategy for indwelling, device-related infections. However, while electrolytically generated chlorine may have antimicrobial properties, as we have demonstrated here, it is also likely to have cytotoxic effects in the surrounding host tissue. We do not necessarily advocate the application of direct electric current as a therapeutic strategy. The message from this investigation is that if a direct current is established *in vivo*, where chloride is abundant, the generation of chlorine is an inevitable consequence. The potential for host tissue damage needs to be addressed if electric current therapies are to be considered. In future work, it will be important to be aware of electrochemistry that can lead to free chlorine when considering the application of direct current technologies *in vivo*.
